# A study on the value of the relationship between newly occurring microinfarctions after stent-assisted treatment for unruptured intracranial aneurysms and systemic inflammatory immune indices

**DOI:** 10.3389/fneur.2025.1679467

**Published:** 2025-11-26

**Authors:** Yifan Lv, Tao Zhang, Lei Li, Guanda Han, Decai Xu, Zhiquan Jiang, Jian Li

**Affiliations:** Department of Neurosurgery at the First Affiliated Hospital of Bengbu Medical University in Bengbu, Anhui, China

**Keywords:** unruptured intracranial aneurysm, interventional therapy, newly developed micro infarcted lesions, systemic immune inflammatory index, inflammation

## Abstract

**Background:**

A paucity of studies has been conducted on the relationship between new microinfarctions following stent-assisted treatment for unruptured intracranial aneurysms (UIA) and systemic inflammatory indices (SII) derived from complete blood counts (CBC) and clinical outcomes. The objective of this study was to assess the possible relationship between SII and the occurrence of new postoperative infarcts.

**Methods:**

A total of 64 patients who underwent UIA stent-assisted therapy at the Department of Neurosurgery, First Affiliated Hospital of Bengbu Medical University, from January 2024 to July 2025 were selected as the study subjects. Blood tests, systemic inflammatory markers [NMP: (Neutrophil Count* Monocyte Count), PLR: (Platelet count/Lymphocyte count), SII: ((Neutrophil Count*Platelet Count)/Lymphocyte Count)], and cranial MRI-DWI were collected at admission and on the third day post-surgery. Patients were divided into two groups, positive and negative, based on the presence of new microinfarctions.

**Results:**

Among the 64 patients included in the study, 24 (37.5%) MRI-DWI reveals new microinfarcts, while 40 (62.5%) MRI-DWI showed no evidence of new microinfarcts. A comparative analysis of the third day postoperatively reveals that white blood cell count, neutrophil count, PLR, and SII increased, while lymphocyte count decreased. The neutrophil count, lymphocyte count, PLR, and SII on the third day postoperatively were found to be associated with the occurrence of new microinfarctions. Third-day SII was independently correlated with new microinfarctions that occurred after UIA stent-assisted therapy.

**Conclusion:**

SII may serve as a potential indicator of the association between postoperative inflammatory response and microinfarction events, with elevated postoperative SII levels correlating with the occurrence of new microinfarctions following UIA stenting procedures.

## 1 Introduction

Intracranial aneurysm (IA) is defined as an abnormal pathological dilation occurring in the wall of intracranial arteries. Clinically, it is generally classified into two categories: unruptured intracranial aneurysm (UIA) and ruptured intracranial aneurysm (RIA). Given the risk of rupture and subsequent hemorrhage associated with unruptured intracranial aneurysm, it is notable that 85% of spontaneous subarachnoid hemorrhages (SAH) are caused by ruptured intracranial aneurysms. Intracranial aneurysm are among the most devastating neurovascular emergencies. The broad patient population and high rates of disability and mortality significantly impact human health and increase family burdens. Consequently, the majority of patients diagnosed with unruptured intracranial aneurysm typically undergo clinical treatment (1).

Advances in medical technology have led to the emergence of stent-assisted coil embolization (SACE) as a significant treatment modality for unruptured intracranial aneurysms (2–4). Nevertheless, thrombus-induced infarction remains a prevalent complication following stent-assisted procedures (5). The majority of postoperative microinfarctions do not manifest with focal neurological deficits and are thus classified as silent embolic infarction (SEI) (6–8). Although most patients with microinfarcts are not afflicted with significant clinical manifestations, they have been identified as risk factors for cognitive decline and dementia onset (9–11). The resulting dementia and cognitive impairment can have deleterious effects on families, communities, and healthcare systems worldwide (12, 13). In recent years, the application of antiplatelet drugs and related genetic testing, thromboelastography testing, and other methods has been found to have predictive value for asymptomatic embolism (14–18), however, the findings of contemporary research are inconclusive, and there is an absence of effective indicators capable of accurately predicting the occurrence of asymptomatic embolism. The systemic immune-inflammatory index [SII: ((Neutrophil Count*Platelet Count)/Lymphocyte Count)], a marker of inflammation, has been found to correlate with the prognosis and onset of cardiovascular diseases, metabolic disorders, and stroke. Its simplicity in acquisition and potential reduction in patient healthcare costs have contributed to its growing recognition (19–21). However, in the domain of cerebrovascular disease research, particularly concerning its association with new microinfarctions following stent-assisted embolization of unruptured intracranial aneurysms, there is a paucity of documented reports. Therefore, the objective of this study is to investigate the correlation between postoperative SII levels and the occurrence of new microinfarctions through retrospective analysis, thereby evaluating its potential value as a predictive biomarker and providing preliminary evidence for subsequent research.

## 2 Subjects and methods

### 2.1 Study subjects

A retrospective, consecutive cohort study was conducted on patients with unruptured intracranial aneurysms who underwent stent-assisted treatment in the Interventional Neurosurgery Department at the First Affiliated Hospital of Bengbu Medical University between January 2024 and July 2025. The inclusion criteria were as follows: (1) patients who signed the relevant informed consent form; (2) patients diagnosed with intracranial aneurysms via digital subtraction angiography (DSA) who required stent placement during endovascular embolization; (3) patients who underwent cranial magnetic resonance imaging (MRI-DWI) and had their relevant laboratory parameters collected and tested at admission and on the third day postoperatively. Exclusion criteria: (1) Patients diagnosed with acute cerebral infarction based on preoperative examinations; (2) patients with a history of organ hemorrhage within the past 3 months; (3) patients with severe liver, renal, or pulmonary dysfunction, or other contraindications for endovascular treatment; (4) Patients who developed acute thrombosis or aneurysm rupture during surgery; (5) Patients with a history of craniotomy for aneurysm clipping surgery; (6) Patients with Moyamoya disease (MMD) or cerebrovascular malformation (CVM) at the same time; (7) Patients with incomplete clinical records.

#### 2.1.1 General information

A retrospective collection of baseline information has been conducted for all patients undergoing stent-assisted treatment for unruptured intracranial aneurysms. The following factors were considered in the study: age, gender, medical history (hypertension, diabetes, cerebral infarction, coronary heart disease), smoking status (continuous smoking or ≥6 months annually or ≥1 cigarette daily), alcohol consumption (≥1 episode monthly) (22), aneurysm location, and selected treatment stents (Pipeline, Atlas, Enterprise, LEO).

#### 2.1.2 Preoperative medication

All patients with unruptured intracranial aneurysms should initiate routine dual antiplatelet therapy 5 days prior to surgery and continue this regimen postoperatively (23). In the event that genetic testing for clopidogrel reveals drug resistance, the patient should be transitioned to ticagrelor (90 mg twice daily) (24). During surgery, the initiation of initial anticoagulation with heparin (100 U/kg) should be administered immediately following the placement of the intermediate catheter. This is to be followed by maintenance therapy at half the initial dose, administered hourly, until the conclusion of the procedure. Ticagrelor, a potential alternative to clopidogrel, is an antiplatelet agent with a low rate of resistance. It has been demonstrated to be as effective and safe as clopidogrel in treating SACE in patients with unruptured intracranial aneurysms (25). It is important to note that this study did not routinely perform preoperative platelet function testing.

#### 2.1.3 Laboratory and imaging tests

Blood samples were collected from all enrolled patients upon admission and on the third day postoperatively. The following tests were performed: white blood cell count, neutrophil count, lymphocyte count, monocyte count, eosinophil count, basophil count, red blood cell count, red blood cell distribution width, platelet count, mean platelet volume, platelet percentage, systemic immune-inflammatory index (SII), platelet-lymphocyte ratio (PLR), neutrophil and monocyte productand(NMP), preoperative and postoperative cranial magnetic resonance imaging (MRI) with diffusion-weighted imaging (DWI). SII: (Neutrophil count x platelet count) ÷ lymphocyte count.

#### 2.1.4 Prognosis assessment

Clinical Definition of New Microembolic Lesions Following UIA Stent-Assisted Therapy: According to the TOAST (Trial of Org 10,172 in Acute Stroke Treatment) classification, microembolic lesions are defined as high-signal regions on diffusion-weighted imaging (DWI) with a diameter of 15 mm or less (22). Two experienced neurosurgeons and radiologists conducted the study by reviewing all preoperative and postoperative MRI-DWI images to determine if new microembolic lesions occurred. The patients were divided into two groups: negative and positive. The positive group was defined as patients who had no new infarcts on preoperative imaging but showed high signal intensity on MRI-DWI on the third postoperative day. The negative group had no new infarcts on preoperative or postoperative imaging.

### 2.2 Statistical methods

Statistical analysis of the data and graphing were performed using SPSS (version 27.0) and GraphPad Prism (version 10.0) software. For continuous data, the mean ± standard deviation (mean ± SD) was used if the distribution was normal: otherwise, the median (M) and interquartile range (IQR, i.e., *P25–P75*) were used. Count data were presented as frequency (n) and proportion (%). Depending on the data type and distribution characteristics, intergroup comparisons were performed using independent samples *t*-tests, Mann–Whitney U tests (nonparametric rank sum tests), or Fisher’s exact probability tests. Variables with statistically significant differences (*p* < 0.05) underwent univariate and multivariate logistic regression analyses. Stepwise regression was used for variable selection, and odds ratios (OR), 95% confidence intervals (CI), and *p* values were calculated to identify relevant risk factors. A *p*-value of less than 0.05 was used as the criterion for determining statistical significance.

## 3 Results

### 3.1 Baseline characteristics of patients

**Figure 1:**
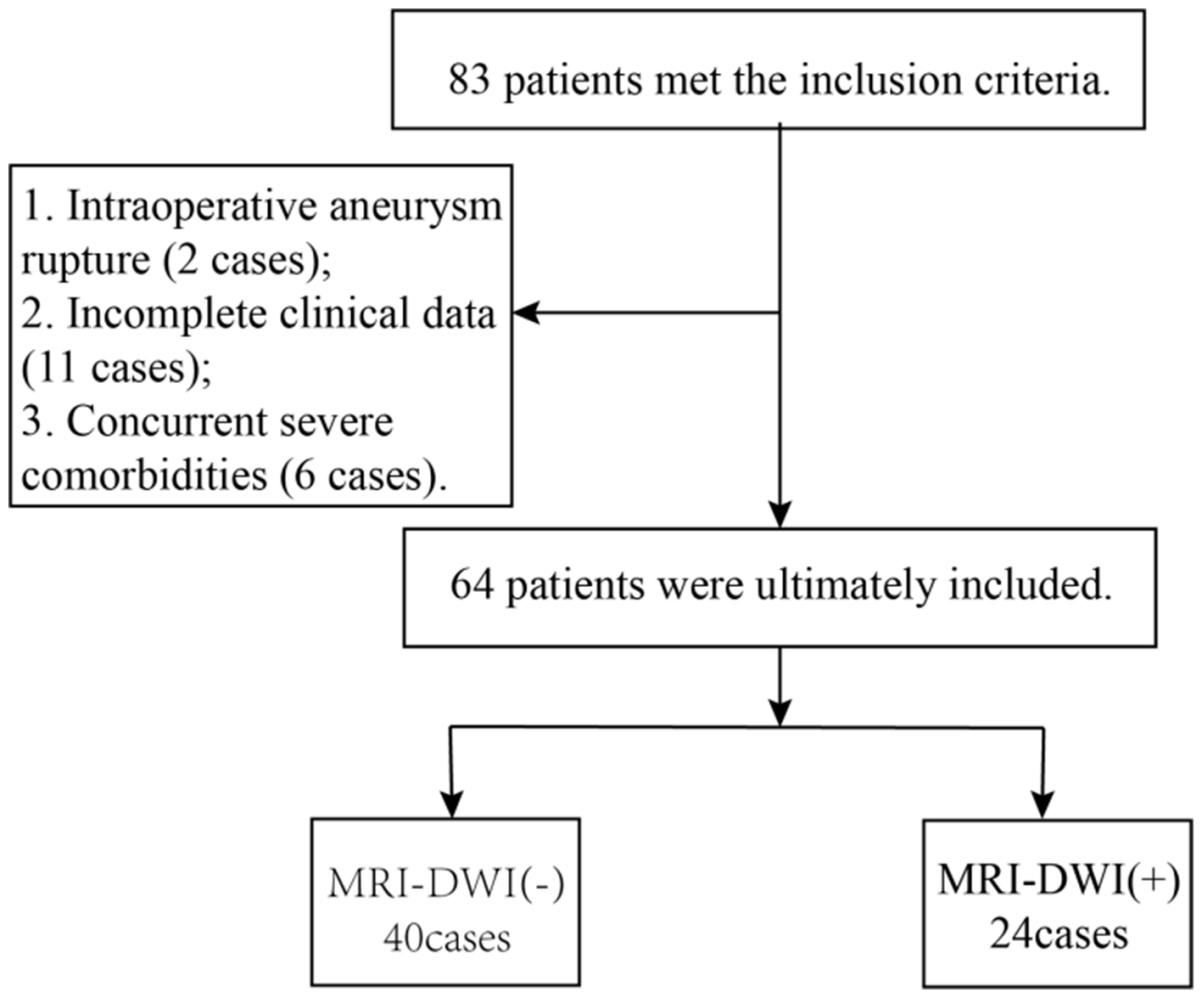
Screening process.

Eighty-three patients who underwent stent-assisted embolization for unruptured intracranial aneurysms were selected based on the inclusion criteria. After applying the exclusion criteria, 19 patients were excluded, leaving 64 patients for the final analysis. The screening process is shown in Figure 1. Baseline clinical characteristics are shown in Table 1. Of the 64 patients, 24 (37.5%) had positive imaging findings and 40 (62.5%) had negative findings. The MRI-DWI (+) group had higher postoperative white blood cell and neutrophil counts, as well as higher PLR and SII, than the MRI-DWI (−) group. These differences were statistically significant (*p* < 0.05). However, the MRI-DWI (+) group had lower postoperative lymphocyte counts than the MRI-DWI (−) group, and these differences were also statistically significant (*p* < 0.05) (see Tables 1–3).

**Table 1:** Comparison of baseline clinical characteristics of patients.

Project	Imaging examination			
	MRI-DWI (−) 40 cases	MRI-DWI (+) 24 cases	Statistic value	*p* value
Age	58.48 ± 12.51	62.96 ± 9.17	*t* = −1.525	0.132
Gender			*χ*^2^ = 2.560	0.110
Men	12 (30.00)	12 (50.00)		
Female	28 (70.00)	12 (50.00)		
Smoking	5 (12.50)	5 (20.83)	*χ*^2^ = 0.284	0.594
Drinking alcohol	3 (7.50)	3 (12.50)	*χ*^2^ = 0.049	0.825
Hypertension	26 (65.00)	18 (75.00)	*χ*^2^ = 0.698	0.403
Diabetes	5 (12.50)	5 (20.83)	*χ*^2^ = 0.284	0.594
Coronary heart disease	1 (2.50)	1 (4.17)	*χ*^2^ = 0.138	0.711
Cerebral infarction	13 (32.50)	12 (50.00)	*χ*^2^ = 1.930	0.165
Treatment stent selection			*χ*^2^ = 3.491	0.386
Pipeline	22 (55.00)	12 (50.00)		
Atlas	8 (20.00)	9 (37.50)		
EP2	8 (20.00)	3 (12.50)		
LEO	2 (5.00)	0 (0.00)		
Location of aneurysm			*χ*^2^ = 4.454	0.486
ICA	10 (25.00)	8 (33.33)		
MCA	2 (5.00)	3 (12.50)		
ACOA	3 (7. 50)	0 (0.00)		
PCOA	6 (15.00)	3 (12.50)		
ICAC6	14 (35.00)	9 (37.50)		
VA	5 (12.50)	1 (4.17)		

ICA, Internal Carotid Artery; MCA, Middle Cerebral Artery; ACOA, Anterior Communicating Artery; PCOA, Posterior Communicating Artery; ICAC6, Ophthalmic Artery; VA, vertebral artery.

**Table 2:** Comparison of preoperative baseline clinical characteristics (complete blood count, inflammatory markers).

Project	Imaging examination			
	MRI-DWI (−) 40 cases	MRI-DWI (+) 24 cases	Statistic value	*p* value
Laboratory Tests				
Complete Blood Count				
White Blood Cell Count ×10^9^/L	6.04 (5.21, 7.14)	5.83 (5.23, 7.30)	*Z* = −0.173	0.862
Monocyte Count ×10^9^/L	0.41 (0.32, 0.60)	0.37 (0.28, 0.41)	*Z* = −1.728	0.084
Lymphocyte Count ×10^9^/L	1.81 (1.42, 2.06)	2.00 (1.50, 2.48)	*Z* = −1.359	0.174
Neutrophil Count ×10^9^/L	3.38 (2.62, 4.16)	3.34 (2.91, 4.46)	*Z* = −0.506	0.613
Eosinophil Count ×10^9^/L	0.15 (0.10, 0.23)	0.13 (0.08, 0.16)	*Z* = −0.937	0.349
Basophil Count ×10^9^/L	0.01 (0.00, 0.02)	0.01 (0.00, 0.02)	*Z* = −0.888	0.375
Red Blood Cell Count ×10^12^/L	4.27 ± 0.43	4.23 ± 0.38	*t* = 0.347	0.730
Red Blood Cell Distribution Width-CV (%)	12.81 ± 0.42	12.93 ± 0.87	*t* = −0.628	0.535
Platelet Count ×10^9^/L	213.00 (198.50, 245.50)	217.50 (190.00, 234.25)	*Z* = −0.458	0.647
Mean Platelet Volume (fL)	10.55 (10.10, 11.03)	10.45 (9.78, 11.45)	*Z* = −0.104	0.917
Platelet Volume Index (mL/L)	0.22 ± 0.04	0.25 ± 0.06	*t* = −1.749	0.085
Inflammatory factors				
NMP	1.42 (0.91, 2.15)	1.20 (0.90, 1.71)	*Z* = −0.945	0.345
PLR	121.29 (102.71, 171.99)	112.54 (87.40, 128.68)	*Z* = −1.588	0.112
SII	451.28 (358.62, 554.32)	379.83 (321.81, 479.84)	*Z* = −1.209	0.227

SII, Systemic immune-inflammatory index; PLR, platelet-lymphocyte ratio; NMP, neutrophil and monocyte productand.

**Table 3:** Comparison of postoperative baseline clinical characteristics (complete blood count, inflammatory markers).

Project	Imaging examination			
	MRI-DWI (−) 40 cases	MRI-DWI (+) 24 cases	Statistic value	*p* value
Laboratory Tests				
Complete blood count				
White Blood Cell Count (10^9^/L)	6.98 (6.18, 9.37)	9.08 (7.63, 10.80)	*Z* = −2.212	0.027
Monocyte Count ×10^9^/L	0.52 (0.43, 0.70)	0.63 (0.43, 0.68)	*Z* = −0.139	0.890
Lymphocyte Count ×10^9^/L	1.69 (1.33, 2.03)	1.02 (0.84, 1.39)	*Z* = −4.348	<0.001
Neutrophil Count ×10^9^/L	4.86 (3.50, 6.75)	7.15 (5.88, 7.87)	*Z* = −3.203	0.001
Eosinophil Count ×10^9^/L	0.07 (0.02, 0.18)	0.03 (0.01, 0.18)	*Z* = −0.703	0.482
Basophil Count ×10^9^/L	0.00 (0.00, 0.01)	0.01 (0.00, 0.02)	*Z* = −0.606	0.544
Red Blood Cell Count ×10^12^/L	3.98 ± 0.44	3.93 ± 0.52	*t* = 0.428	0.670
Red Blood Cell Distribution Width-CV (%)	13.13 ± 0.76	12.93 ± 0.80	*t* = 0.996	0.323
Platelet Count ×10^9^/L	205.50 (176.75, 220.75)	237.50 (185.50, 243.75)	*Z* = −1.221	0.222
Mean Platelet Volume (fL)	10.25(9.90, 10.73)	10.250(9.50, 11.35)	*Z* = −0.215	0.830
Platelet Volume Index (ml/L)	0.22 ± 0.05	0.22 ± 0.05	*t* = −0.153	0.879
Inflammatory factors				
NMP	2.72 (1.61, 4.89)	4.01 (2.53, 4.95)	*Z* = −1.755	0.079
PLR	118.95 (98.73, 160.98)	201.00 (166.43, 245.70)	*Z* = −4.889	<0.001
SII	605.09 (422.36, 895.43)	1390.81 (1143.62, 1751.98)	*Z* = −5.440	<0.001

SII, Systemic immune-inflammatory index; PLR, platelet-lymphocyte ratio; NMP, neutrophil and monocyte productand.

### 3.2 Logistic regression analysis of relevant influencing factors

Variables that showed statistically significant differences in Tables 1–3 were included in the univariate logistic regression analysis. The results of the analysis showed that: Postoperative neutrophil count (*p* = 0.010), postoperative lymphocyte count (*p* < 0.001), postoperative PLR (*p* < 0.001), and postoperative SII (*p* < 0.001) were statistically significant. Variables with *p* < 0.05 in the univariate analysis were included in the multivariate analysis. Variable selection was performed using stepwise regression. The findings of this study suggest that postoperative SII is an independent factor influencing microembolization following stent-assisted repair of unruptured intracranial aneurysms (*p* < 0.05) (see Table 4).

**Table 4:** Logistic regression analysis of correlated factors for postoperative microembolization in stent-assisted repair of unruptured intracranial aneurysms.

Variables	Univariate analysis					Multivariate analysis				
	*β*	S. E	*Z*	*p*	OR (95% CI)	*β*	S. E	*Z*	*p*	OR (95% CI)
Postoperative white blood cell count	0.183	0.097	1.885	0.059	1.201 (0.993 ~ 1.452)					
Postoperative neutrophil count	0.336	0.130	2.577	0.010	1.399 (1.084 ~ 1.807)					
Postoperative lymphocyte count	−2.609	0.774	−3.370	<0.001	0.074 (0.016 ~ 0.336)	−1.213	0.908	−1.336	0.181	0.297 (0.050 ~ 1.761)
Postoperative PLR	0.026	0.007	3.661	<0.001	1.026 (1.012 ~ 1.040)					
Postoperative SII	0.003	0.001	3.872	<0.001	1.003 (1.002 ~ 1.005)	0.003	0.001	2.947	0.003	1.003 (1.001 ~ 1.004)

SII, Systemic immune-inflammatory index; PLR, platelet-lymphocyte ratio; NMP, neutrophil and monocyte productand.

### 3.3 ROC curve analysis of the correlation between different indicators and new-onset microinfarction following stent-assisted embolization of unruptured intracranial aneurysms

**Figure 2:**
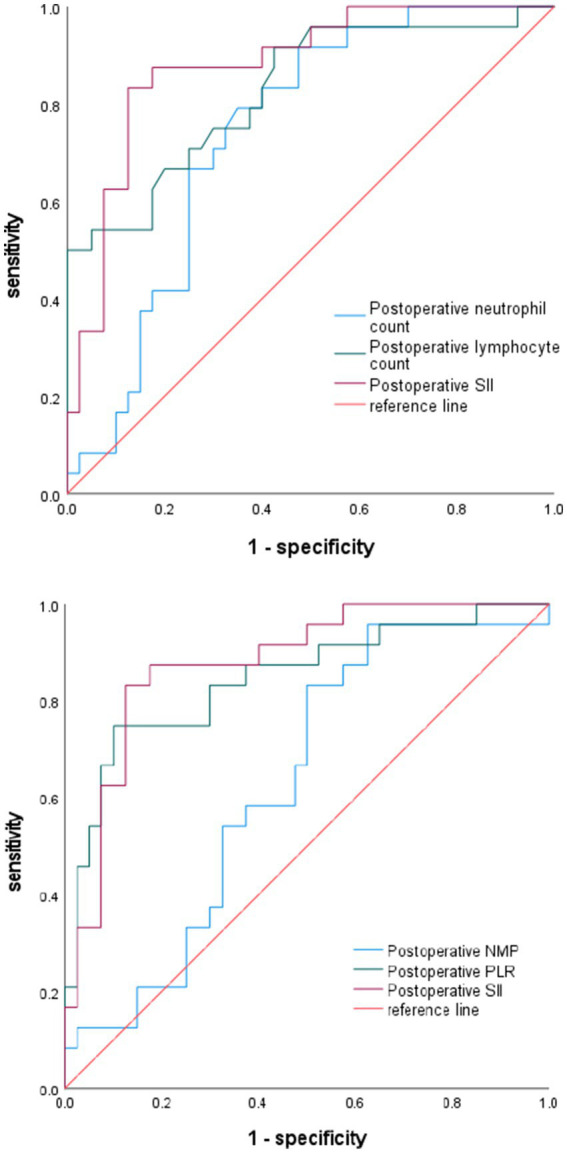
Diagnostic value of neutrophil count, lymphocyte count, NMP, PLR, and SII for postoperative infarction.

The diagnostic specificity of SII was assessed by plotting its ROC curve and comparing it with postoperative neutrophil count, postoperative lymphocyte count, postoperative NMP, and postoperative PLR. The findings indicated that the SII effectively predicted new microinfarction following stent-assisted treatment for unruptured intracranial aneurysms, with an area under the curve (AUC) of 0.879 [95% confidence interval (CI): 0.793–0.965, *p* < 0.0001]. The optimal cutoff value for the SII was determined to be 1086.147, with sensitivity and specificity values of 83.3 and 87.5%, respectively. SII exhibited superior predictive accuracy for post-operative new-onset microinfarction in comparison to post-operative neutrophil count (AUC 0.879 vs. 0.741, *p* = 0.008), as well as post-operative lymphocyte count (AUC 0.879 vs. 0.827, *p* = 0.329). Additionally, SII demonstrated comparable predictive accuracy for postoperative PLR (AUC 0.879 vs. 0.847, *p* = 0.412). Postoperative NMP demonstrated no substantial predictive capacity for new-onset microinfarction following stent-assisted treatment of unruptured intracranial aneurysms (AUC 0.632, *p* = 0.0782) (Figure 2; Table 5).

**Table 5:** The correlational value of relevant influencing factors on postoperative microinfarction.

Variables	*AUC*	Best cut-off value	Youden index	95% CI	Sensitivity%	Specificity%	*p* value
Postoperative neutrophil count	0.741	5.825	0.442	0.621–0.861	79.2%	65%	0.0013
Postoperative lymphocyte count	0.827	1.040	0.492	0.720–0.934	95%	55%	<0.0001
Postoperative NMP	0.632	2.429	0.333	0.495–0.770	83.3%	50%	0.0782
Postoperative PLR	0.847	169.625	0.650	0.743–0.951	75%	90%	<0.0001
Postoperative SII	0.879	1086.147	0.708	0.793–0.965	83.3%	87.5%	<0.0001

SII, Systemic immune-inflammatory index; PLR, platelet-lymphocyte ratio; NMP, neutrophil and monocyte productand.

## 4 Clinical cases

**Figure 3:**
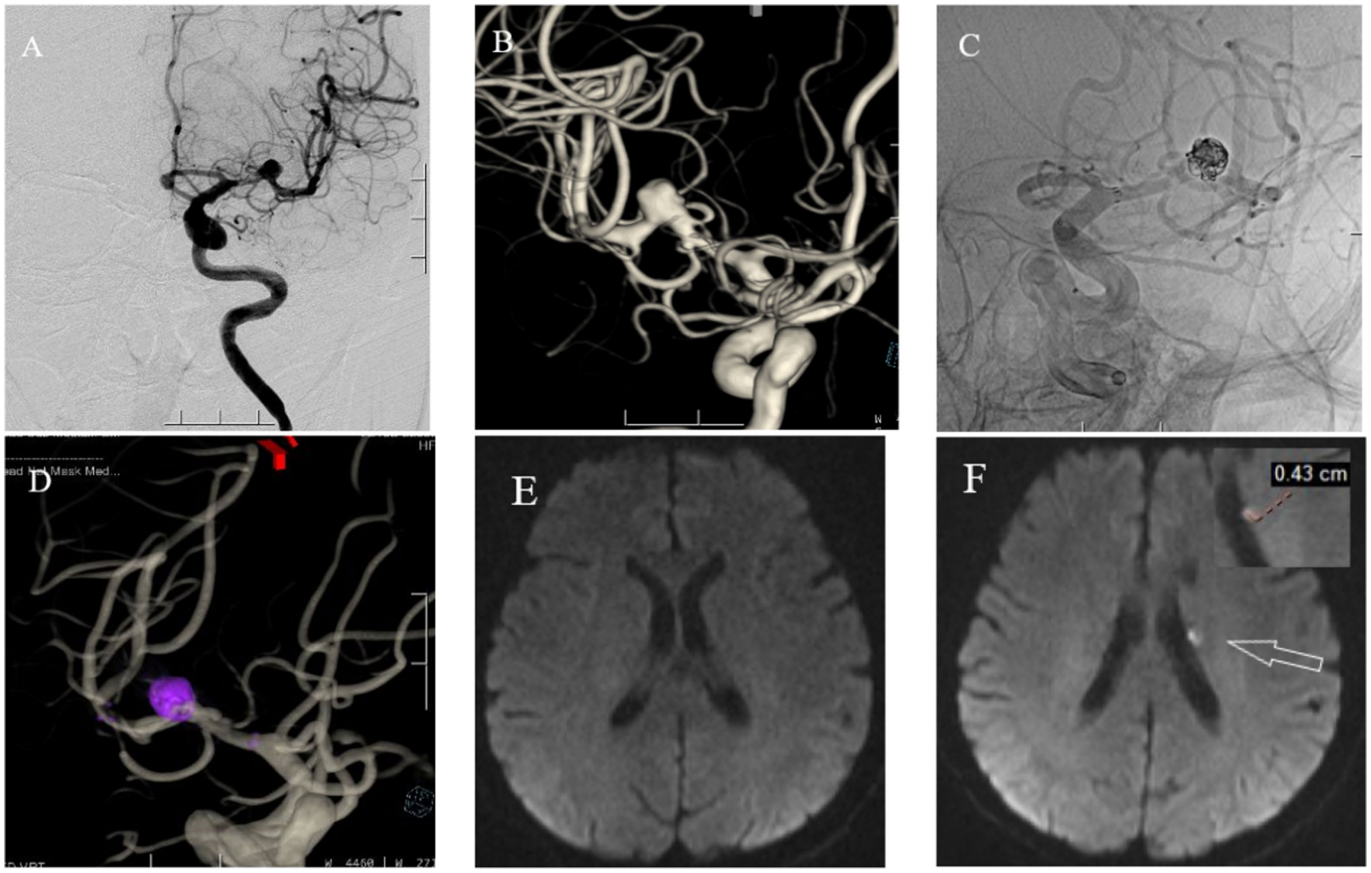
A 71 year old female with headaches, dizziness, and transient fainting for half a month. **(A)** Cranial DSA showing the location of the intracranial aneurysm in the 2D plane during the operation. **(B)** Cranial DSA showing a 3D image of the left middle cerebral artery aneurysm. **(C)** Intraoperative 2D plane showing the placement of coils and stents. **(D)** Postoperative 3D plane showing the placement of coils and stents. **(E)** Preoperative MRI-DWI showed no new infarcts. **(F)** Postoperative MRI-DWI on day three showed an acute microinfarction lesion in the left periventricular region with a diameter of approximately 0.43 cm.

**Figure 4:**
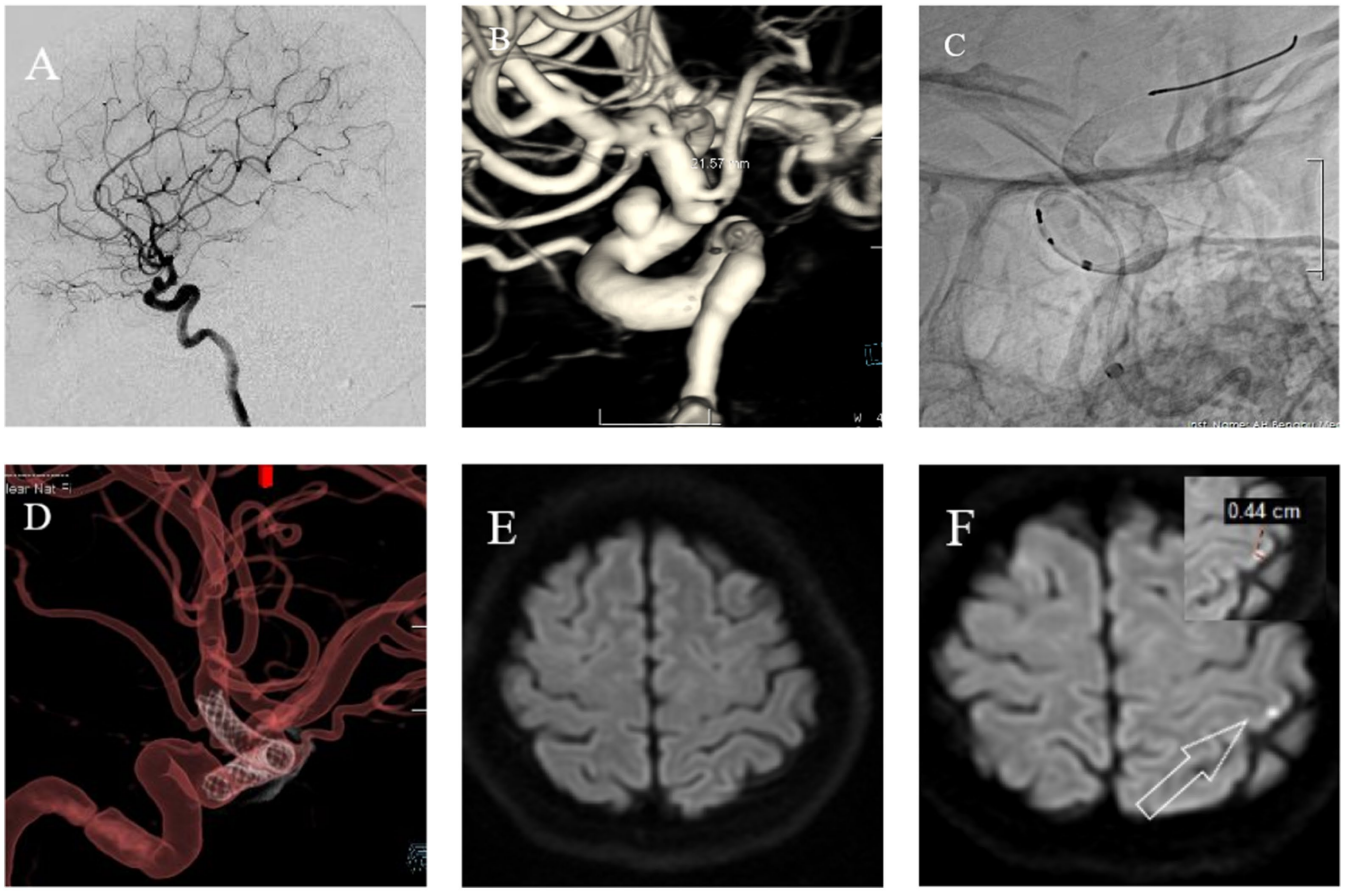
Female, 56 years old. Intermittent headaches for over 7 months, worsening for 1 week. **(A)** Cranial DSA showing the location of the intracranial aneurysm in the 2D plane during the operation. **(B)** Cranial DSA showing the location of the aneurysm in the 3D image, confirming it is in the ophthalmic artery segment of the left internal carotid artery. **(C)** Intraoperative 2D plane showing the location of the stent. **(D)** Postoperative 3D plane showing the stent model. **(E)** Preoperative MRI-DWI showed no new infarcts. **(F)** Postoperative day 3 MRI-DWI showed an acute microinfarction lesion in the left parietal lobe with a diameter of approximately 0.44 cm.

The following brief descriptions of cases of postoperative microembolic infarction are based on clinical treatment and imaging data, as shown in Figures 3, 4.

## 5 Discussion

The present study found that among patients undergoing endovascular treatment for unruptured intracranial aneurysms, the incidence of new microinfarcts postoperatively was 37.5%. The overwhelming majority of cases (95.8%) were characterized by the presence of new asymptomatic microembolic lesions, with only 4.2% of cases demonstrating associated neurological deficits. This finding is consistent with the study by Park et al. (26). Although most patients with microinfarcts are not afflicted with significant clinical manifestations, they may increase the risk of long-term ischemic stroke, vascular cognitive impairment, and neuropsychological dysfunction as a specific type of ischemic brain injury. Microinfarcts have been identified as a risk factor for cognitive decline and dementia (11, 27, 28).

The study primarily obtained patients’ admission and postoperative hematological laboratory data, as blood biomarkers are widely used for disease prediction and are readily available and cost-effective in most medical institutions, thereby guiding disease diagnosis and monitoring prognosis. A significant proportion of biomarkers are classified as inflammatory markers. Inflammation plays a pivotal role in the pathophysiology preceding and following infarction. Abnormal immune responses that result in inflammatory changes to the vascular wall form the basis of atherosclerotic plaques, which are a precursor to ischemic stroke (29). Inflammatory response is a common reaction to any local tissue injury. It is widely accepted that hemodynamic stress functions as the primary catalyst for the initiation of inflammatory processes within the arterial wall. These processes are initiated by the infiltration of various inflammatory cells, including monocytes, neutrophils, and lymphocytes. These cells undergo further differentiation, synthesize inflammatory cytokines and immunoglobulins, and activate the complement system. Furthermore, vascular inflammation results in altered endothelial function, disruption of the internal elastic lamina and collagen matrix, and activation and proliferation of vascular endothelial cells. This, in turn, has been shown to promote oxidative stress, cell adhesion, and intraluminal thrombosis (30). At present, the role of complete blood cell counts in predicting the prognosis of patients with new-onset myocardial infarction and cerebral infarction is often underestimated by clinicians and patients alike. Moreover, there remains a paucity of effective blood biomarkers for prognostic assessment. In comparison with single markers, composite inflammation indices that incorporate multiple indicators are believed to provide a more comprehensive view of inflammation.

A number of studies have indicated that neutrophils and monocytes function as mediators of innate immunity and inflammatory responses, while lymphocytes are implicated in adaptive immune responses (31–33). Neutrophils and monocytes are typically regarded as deleterious, whereas lymphocytes are predominantly considered to have a protective effect on outcomes following myocardial infarction (34). Neutrophils have been shown to play a pivotal role in the inflammatory response of atherosclerosis by secreting substantial amounts of inflammatory mediators, such as IL-1, IL-6, TNF-α, and oxygen free radicals. These pro-inflammatory and destructive mediators promote cellular damage, damage to the extracellular matrix and blood–brain barrier, and ultimately lead to secondary brain injury (32, 33). Lymphocytes have been identified as the primary neuroprotective immune modulators, exhibiting the capacity to inhibit various inflammatory pathways and to reduce the activation and recruitment of both resident and invading immune cells (35). However, lymphocytes have been shown to generate specific immune responses against oxidized low-density lipoprotein (ox-LDL) and to be activated and differentiated into Th1 cells, which secrete large amounts of pro-inflammatory cytokines such as interleukin-2 (IL-2). Interleukin-3 (IL-3), tumor necrosis factor-α (TNF-α), and interferon-γ have been identified as key factors that activate macrophages, endothelial cells, and vascular smooth muscle cells, thereby exacerbating local inflammation and promoting the progression of atherosclerosis (35, 36). Moreover, platelets have been demonstrated to modulate hemostasis, thrombosis, and inflammation (37). The activation and adhesion of platelets on damaged endothelium, as well as platelet-leukocyte interactions, are important pathophysiological processes in thrombotic inflammation that can lead to cerebral vascular injury (38, 39).

Recent studies have also reported that an increase in neutrophils is associated with adverse short-term outcomes, suggesting that acute local neutrophil-dominant inflammation plays a role (40). In this study, we observed no significant differences in hematological indicators such as white blood cell count, monocyte count, lymphocyte count, neutrophil count, NMP, PLR, and SII between preoperative MRI-DWI (−) groups and preoperative MRI-DWI (+) groups. A comparison of the postoperative MRI-DWI (+) group and the preoperative MRI-DWI (+) group revealed that the former had significantly higher white blood cell counts, neutrophil counts, platelet-to-lymphocyte ratio (PLR), and systemic inflammation index (SII) (*p* < 0.05). However, the lymphocyte count in the postoperative MRI-DWI (+) group was lower than that in the preoperative MRI-DWI (+) group (*p* < 0.05). It was observed that the hematological parameters, including white blood cell count, lymphocyte count, neutrophil count, platelet-to-lymphocyte ratio (PLR), and systemic immune-inflammation index (SII), on the third day postoperatively were associated with the occurrence of new microinfarctions postoperatively, which was consistent with the findings of Cao et al. (41). The results of this study suggest a potential harmful effect of white blood cells and neutrophils, and a lack of brain protective effects associated with lymphocyte deficiency, which may contribute to adverse outcomes following unruptured intracranial aneurysms surgery.

NMP, PLR and SII are innovative biomarkers initially developed for cancer research to assess thrombotic inflammatory responses. In recent years, these markers have also demonstrated clinical significance in cardiovascular diseases. A small-scale observational study indicated that the novel inflammatory markers PLR and SII are associated with the risk of carotid atherosclerosis in middle-aged and elderly men (41, 42). The findings of this study revealed that there was no statistically significant difference in inflammatory factor levels between the preoperative MRI-DWI (+) group and the MRI-DWI (−) group. However, patients in the MRI-DWI (+) group exhibited significantly elevated inflammatory factor levels (PLR, SII) in comparison to those in the MRI-DWI (−) group postoperatively. Moreover, patients in the MRI-DWI (+) group demonstrated significantly higher inflammatory factor levels postoperatively than those in the MRI-DWI (+) group prior to surgery. The present study demonstrates an independent association between PLR and SII on postoperative day 3 and the occurrence of new microinfarctions following stent-assisted treatment of unruptured intracranial aneurysms. This finding is consistent with the results reported by Göçmen et al. (43). In light of these findings, we hypothesize that PLR and SII may serve as promising inflammatory biomarkers associated with this postoperative complication. In light of the current paucity of exploration of inflammatory markers for postoperative embolization events in neurointerventional fields, the execution of rigorously designed prospective studies is imperative to substantiate their prospective value in prognostic assessment. This study provides significant preliminary evidence, thereby establishing the research value of SII as an inflammatory marker associated with postoperative microinfarction.

The limitations of this study are as follows: (1) As a single-center retrospective study, it inherently possesses limitations that may compromise statistical power, potentially restricting the generalizability of findings to broader populations. (2) The study did not routinely perform preoperative platelet function testing, as the results are susceptible to various preanalytical factors, including blood collection techniques, sample transport time, and testing method standardization (44, 45). In order to ensure consistency in the interpretation of data for all enrolled patients and to avoid additional confounding factors arising from inconsistent testing conditions, PFTs were not systematically performed in this study. Consequently, the interpretation of the association between postoperative SII and microinfarction should be considered exploratory, and its clinical translation requires further validation in prospective studies with standardized platelet function testing. 3. Although MRI-DWI was employed to identify acute ischemic lesions, a systematic quantitative analysis of lesion volume and number was lacking. 4. Due to the limited sample size, a comparative analysis of treatment stent differences was not conducted. 5. Due to the limited sample size, the study may not fully reflect the diversity of predictive indicators. Future research should involve multicenter, large-scale data to exclude relevant confounding factors, ensuring higher reliability and broader applicability of the findings.

## 6 Conclusion

The present study found that elevated SII levels after UIA stent-assisted therapy were independently associated with the occurrence of new microinfarctions postoperatively. It is imperative to underscore that this finding emanates from a retrospective analysis that compares preoperative and postoperative data. The findings of this study suggest that SII may serve as a potential indicator of an association between postoperative inflammatory response and microinfarction events, rather than a clinical tool with prospective predictive capability. The current data set is inadequate to substantiate its direct application in modifying clinical management or improving patient outcomes. To further substantiate the clinical significance of SII, future prospective studies with meticulous control of confounding factors are imperative.

## Data Availability

The original contributions presented in the study are included in the article/supplementary material, further inquiries can be directed to the corresponding author.

## References

[1] Tawk RG Hasan TF D'souza CE Peel JB Freeman WD. Diagnosis and treatment of unruptured intracranial aneurysms and aneurysmal subarachnoid hemorrhage. Mayo Clin Proc. (2021) 96:1970–2000. doi: 10.1016/j.mayocp.2021.01.005, PMID: 33992453

[2] Kim D Chung J. Y-stent-assisted coiling with neuroform atlas stents for wide-necked intracranial bifurcation aneurysms: a preliminary report. J Cerebrovasc Endovasc Neurosurg. (2022) 24:1–9. doi: 10.7461/jcen.2021.E2021.06.010 34579507 PMC8984637

[3] Han JT Zhang YX Jia ZC Jiang CH Liu L Luan JY . Clinical application of Neuroform atlas stent-assisted coiling in the treatment of unruptured wide-neck intracranial aneurysms. Beijing Da Xue Xue Bao Yi Xue Ban. (2023) 55:139–43. doi: 10.19723/j.issn.1671-167X.2023.01.021 36718702 PMC9894790

[4] Zaidat OO Hanel RA Sauvageau EA Aghaebrahim A Lin E Jadhav AP . Pivotal trial of the neuroform atlas stent for treatment of anterior circulation aneurysms: one-year outcomes. Stroke. (2020) 51:2087–94. doi: 10.1161/STROKEAHA.119.028418, PMID: 32568654 PMC7306258

[5] Habtezghi AB Ghozy S Bilgin C Kobeissi H Kadirvel R Kallmes DF. DWI-detected ischemic lesions after endovascular treatment for cerebral aneurysms: an updated systematic review and meta-analysis. AJNR Am J Neuroradiol. (2023) 44:1256–61. doi: 10.3174/ajnr.A8024, PMID: 37827721 PMC10631525

[6] Bond KM Brinjikji W Murad MH Kallmes DF Cloft HJ Lanzino G. Diffusion-weighted imaging-detected ischemic lesions following endovascular treatment of cerebral aneurysms: a systematic review and meta-analysis. AJNR Am J Neuroradiol. (2017) 38:304–9. doi: 10.3174/ajnr.A4989, PMID: 27856436 PMC7963818

[7] Suzuki R Takigawa T Nagaishi M Hyodo A Suzuki K. Global outflow angle influences silent ischemic events in coil embolization for unruptured distal anterior cerebral artery aneurysms. Interv Neuroradiol. (2024) 30:72–9. doi: 10.1177/15910199221104915, PMID: 35635226 PMC10956461

[8] Shin S Hwangbo L Lee TH Ko JK. Silent embolic infarction after Neuroform atlas stent-assisted coiling of unruptured intracranial aneurysms. J Korean Neurosurg Soc. (2024) 67:42–9. doi: 10.3340/jkns.2023.0091, PMID: 37661088 PMC10788554

[9] Decarli C. Clinically asymptomatic vascular brain injury: a potent cause of cognitive impairment among older individuals. J Alzheimer's Dis. (2013) 33:S417–26. doi: 10.3233/JAD-2012-129004 23034523 PMC3786369

[10] Goldberg I Auriel E Russell D Korczyn AD. Microembolism, silent brain infarcts and dementia. J Neurol Sci. (2012) 322:250–3. doi: 10.1016/j.jns.2012.02.021, PMID: 22429666

[11] Durrani R Hill MD Smith EE. Preventing covert brain infarct-related cognitive impairment and dementia. Can J Neurol Sci. (2020) 47:456–63. doi: 10.1017/cjn.2020.45 32122431

[12] GBD 2019 Dementia Forecasting Collaborators. Estimation of the global prevalence of dementia in 2019 and forecasted prevalence in 2050: an analysis for the global burden of disease study 2019. Lancet Public Health. (2022) 7:e105–25. doi: 10.1016/S2468-2667(21)00249-8 34998485 PMC8810394

[13] GBD 2016 Dementia Collaborators. Global, regional, and national burden of Alzheimer's disease and other dementias, 1990-2016: a systematic analysis for the global burden of disease study 2016. Lancet Neurol. (2019) 18:88–106. doi: 10.1016/S1474-4422(18)30403-4 30497964 PMC6291454

[14] Xu R Cheng C Wu Y Guo Z Sun X Xia Y . Microbleeds after stent-assisted coil embolization of unruptured intracranial aneurysms: incidence, risk factors and the role of thromboelastography. Curr Neurovasc Res. (2020) 17:502–9. doi: 10.2174/1567202617999200819161033, PMID: 32814525

[15] Yang H Li Y Jiang Y Lv X. Thromboelastography for monitoring platelet function in unruptured intracranial aneurysm patients undergoing stent placement. Interv Neuroradiol. (2015) 21:61–8. doi: 10.15274/INR-2014-10094, PMID: 25934777 PMC4757209

[16] Wang X Luo L Wang Y An Z. Effect of platelet function testing guidance on clinical outcomes for patients with intracranial aneurysms undergoing endovascular treatment. AJNR Am J Neuroradiol. (2023) 44:928–33. doi: 10.3174/ajnr.A7923, PMID: 37414457 PMC10411848

[17] Kim GJ Heo Y Moon EJ Park W Ahn JS Lee DH . Thromboembolic events during endovascular coiling for unruptured intracranial aneurysms: clinical significance of platelet reactivity unit and adjunctive cilostazol. Clin Neurol Neurosurg. (2022) 213:107133. doi: 10.1016/j.clineuro.2022.107133, PMID: 35065532

[18] Li W Wang A Ma C Wang Y Zhao Y Zhang Y . Antiplatelet therapy adjustment improved the radiomic characteristics of acute silent cerebral infarction after stent-assisted coiling in patients with high on-treatment platelet reactivity: a prospective study. Front Neurosci. (2023) 17:1068047. doi: 10.3389/fnins.2023.1068047, PMID: 36845416 PMC9948085

[19] Tian A Zheng Y Jin J Huang C. Association of systemic inflammatory response index and stroke: a cross-sectional study of NHANES, 2005-2018. Front Neurol. (2025) 16:1538352. doi: 10.3389/fneur.2025.1538352, PMID: 39958615 PMC11825461

[20] Wang Q Li WN Otkur W Cui Y Chen HS. Neutrophil-to-lymphocyte ratio, platelet-to-lymphocyte ratio, systemic immune inflammation index and efficacy of remote ischemic conditioning in acute ischemic stroke: a post hoc exploratory analysis of the RICAMIS study. J Inflamm Res. (2024) 17:5543–53. doi: 10.2147/JIR.S460928, PMID: 39185106 PMC11344552

[21] Liu B Wang J Li YY Li KP Zhang Q. The association between systemic immune-inflammation index and rheumatoid arthritis: evidence from NHANES 1999-2018. Arthritis Res Ther. (2023) 25:34. doi: 10.1186/s13075-023-03018-6, PMID: 36871051 PMC9985219

[22] Shi Y Guo L Chen Y Xie Q Yan Z Liu Y . Risk factors for ischemic stroke: differences between cerebral small vessel and large artery atherosclerosis aetiologies. Folia Neuropathol. (2021) 59:378–85. doi: 10.5114/fn.2021.112007, PMID: 35114778

[23] Meyer BM Campos JK Collard De Beaufort JC Chen I Khan MW Amin G . Trends in dual antiplatelet therapy use for neurointerventional procedures for the management of intracranial aneurysms. Biomedicine. (2023) 11:2234. doi: 10.3390/biomedicines11082234, PMID: 37626730 PMC10452183

[24] Mohammaden MH English SW Stapleton CJ Khedr E Shoyb A Hegazy A . Safety and efficacy of ticagrelor as single antiplatelet therapy in prevention of thromboembolic complications associated with the pipeline embolization device (PED): multicenter experience. J Neurointerv Surg. (2020) 12:1113–6. doi: 10.1136/neurintsurg-2020-015978, PMID: 32471826

[25] Park KY Ozaki T Kostynskyy A Kortman H Hilario A Nicholson P . Ticagrelor versus clopidogrel in the dual antiplatelet regimen for intracranial stenting or flow-diverter treatment for unruptured cerebral aneurysms: a single-center cohort study. AJNR Am J Neuroradiol. (2021) 42:1638–44. doi: 10.3174/ajnr.A7216, PMID: 34244132 PMC8423043

[26] Park KY Kim BM Kim DJ Kim DI Heo JH Nam HS . Incidence and risk factors for diffusion-weighted imaging (+) lesions after intracranial stenting and its relationship with symptomatic ischemic complications. Stroke. (2014) 45:3298–303. doi: 10.1161/STROKEAHA.114.006182, PMID: 25300970

[27] Paraskevas KI Mikhailidis DP Spinelli F Faggioli G Saba L Silvestrini M . Asymptomatic carotid stenosis and cognitive impairment. J Cardiovasc Surg. (2023) 64:167–73. doi: 10.23736/S0021-9509.23.12620-6, PMID: 36790142

[28] Hilal S Doolabi A Vrooman H Ikram MK Ikram MA Vernooij MW. Clinical relevance of cortical cerebral microinfarcts on 1.5T magnetic resonance imaging in the late-adult population. Stroke. (2021) 52:922–30. doi: 10.1161/STROKEAHA.120.032085 33535785

[29] Camps-Renom P Mccabe J MARTí-FàBREGAS J Giannotti N Fernández-León A McNulty JP . Association of plaque inflammation with stroke recurrence in patients with unproven benefit from carotid revascularization. Neurology. (2022) 99:e109–18. doi: 10.1212/WNL.0000000000200525, PMID: 35418461

[30] Jabbarli R Rauschenbach L Dinger TF Darkwah Oppong M Rodemerk J Pierscianek D . In the wall lies the truth: a systematic review of diagnostic markers in intracranial aneurysms. Brain Pathol. (2020) 30:437–45. doi: 10.1111/bpa.12828, PMID: 32068920 PMC8017992

[31] Szepanowski RD Haupeltshofer S Vonhof SE Frank B Kleinschnitz C Casas AI. Thromboinflammatory challenges in stroke pathophysiology. Semin Immunopathol. (2023) 45:389–410. doi: 10.1007/s00281-023-00994-4, PMID: 37273022 PMC10241149

[32] Carmona-Mora P Knepp B Jickling GC Zhan X Hakoupian M Hull H . Monocyte, neutrophil, and whole blood transcriptome dynamics following ischemic stroke. BMC Med. (2023) 21:65. doi: 10.1186/s12916-023-02766-1, PMID: 36803375 PMC9942321

[33] Simats A Liesz A. Systemic inflammation after stroke: implications for post-stroke comorbidities. EMBO Mol Med. (2022) 14:e16269. doi: 10.15252/emmm.202216269, PMID: 35971650 PMC9449596

[34] Liu R Song P Gu X Liang W Sun W Hua Q . Comprehensive landscape of immune infiltration and aberrant pathway activation in ischemic stroke. Front Immunol. (2021) 12:766724. doi: 10.3389/fimmu.2021.766724, PMID: 35140708 PMC8818702

[35] Xiao J Qiu QW Qin C Tao R Qiao SY Chen M . Dynamic changes of peripheral blood lymphocyte subsets in acute ischemic stroke and prognostic value. Brain Behav. (2021) 11:e01919. doi: 10.1002/brb3.1919, PMID: 33111494 PMC7821621

[36] Li S Huang Y Liu Y Rocha M Li X Wei P . Change and predictive ability of circulating immunoregulatory lymphocytes in long-term outcomes of acute ischemic stroke. J Cereb Blood Flow Metab. (2021) 41:2280–94. doi: 10.1177/0271678X21995694, PMID: 33641517 PMC8393304

[37] Sharma S Tyagi T Antoniak S. Platelet in thrombo-inflammation: unraveling new therapeutic targets. Front Immunol. (2022) 13:1039843. doi: 10.3389/fimmu.2022.1039843, PMID: 36451834 PMC9702553

[38] Denorme F Ajanel A Campbell RA. Immunothrombosis in neurovascular disease. Res Pract Thromb Haemost. (2024) 8:102298. doi: 10.1016/j.rpth.2023.102298, PMID: 38292352 PMC10825058

[39] De Meyer SF Langhauser F Haupeltshofer S Kleinschnitz C Casas AI. Thromboinflammation in brain ischemia: recent updates and future perspectives. Stroke. (2022) 53:1487–99. doi: 10.1161/STROKEAHA.122.038733, PMID: 35360931

[40] Kollikowski AM Pham M März AG Papp L Nieswandt B Stoll G . Platelet activation and chemokine release are related to local neutrophil-dominant inflammation during hyperacute human stroke. Transl Stroke Res. (2022) 13:364–9. doi: 10.1007/s12975-021-00938-w, PMID: 34455571 PMC9046342

[41] Cao W Song Y Bai X Yang B Li L Wang X . Systemic-inflammatory indices and clinical outcomes in patients with anterior circulation acute ischemic stroke undergoing successful endovascular thrombectomy. Heliyon. (2024) 10:e31122. doi: 10.1016/j.heliyon.2024.e31122, PMID: 38778990 PMC11109896

[42] Chen Q Che M Shen W Shao L Yu H Zhou J. Comparison of the early warning effects of novel inflammatory markers SIRI, NLR, and LMR in the inhibition of carotid atherosclerosis by testosterone in middle-aged and elderly Han Chinese men in the real world: a small sample clinical observational study. Am J Mens Health. (2023) 17:15579883231171462. doi: 10.1177/15579883231171462, PMID: 37183913 PMC10186581

[43] GöçMEN A Gesoglu Demir T. The aggregate index of systemic inflammation as a predictor of mortality in stroke patients. Cureus. (2024) 16:e64007. doi: 10.7759/cureus.64007 39109115 PMC11301770

[44] Frelinger AL III Rivera J Connor DE Freson K Greinacher A Harrison P. Consensus recommendations on flow cytometry for the assessment of inherited and acquired disorders of platelet number and function: communication from the ISTH SSC subcommittee on platelet physiology. J Thromb Haemost. (2021) 19:3193–202. doi: 10.1111/jth.15526 34580997

[45] Denessen EJS Van Den Kerkhof DL Jeurissen MLJ Wetzels RJH Verhezen PWM Henskens YMC. Determining the optimal storage time and temperature for performing platelet function assays and global hemostasis assays. Platelets. (2022) 33:416–24. doi: 10.1080/09537104.2021.1934666 34115551

